# Genetic susceptibility and causal pathway analysis of eye disorders coexisting in multiple sclerosis

**DOI:** 10.3389/fimmu.2024.1337528

**Published:** 2024-02-05

**Authors:** Xuecheng Qiu, Mi Ni Huang, Suning Ping

**Affiliations:** ^1^ Jiangsu Key Laboratory of Brain Disease Bioinformation, Research Center for Biochemistry and Molecular Biology, Xuzhou Medical University, Xuzhou, Jiangsu, China; ^2^ Molecular Cancer Research Center, School of Medicine, Shenzhen Campus of Sun Yat-Sen University, Shenzhen, Guangdong, China; ^3^ Department of Histology and Embryology, School of Medicine, Shenzhen Campus of Sun Yat-Sen University, Shenzhen, Guangdong, China; ^4^ Neurobiology Research Center, School of Medicine, Shenzhen Campus of Sun Yat-Sen University, Shenzhen, Guangdong, China

**Keywords:** Mendelian randomization, genetic susceptibility, causal pathway, multiple sclerosis, eye disorder, innate immunity

## Abstract

**Introduction:**

The comorbidity of optic neuritis with multiple sclerosis has been well recognized. However, the causal association between multiple sclerosis and optic neuritis, as well as other eye disorders, remains incompletely understood. To address these gaps, we investigated the genetically relationship between multiple sclerosis and eye disorders, and explored potential drugs.

**Methods:**

In order to elucidate the genetic susceptibility and causal links between multiple sclerosis and eye disorders, we performed two-sample Mendelian randomization analyses to examine the causality between multiple sclerosis and eye disorders. Additionally, causal single-nucleotide polymorphisms were annotated and searched for expression quantitative trait loci data. Pathway enrichment analysis was performed to identify the possible mechanisms responsible for the eye disorders coexisting with multiple sclerosis. Potential therapeutic chemicals were also explored using the Cytoscape.

**Results:**

Mendelian randomization analysis revealed that multiple sclerosis increased the incidence of optic neuritis while reducing the likelihood of concurrent of cataract and macular degeneration. Gene Ontology enrichment analysis implicated that lymphocyte proliferation, activation and antigen processing as potential contributors to the pathogenesis of eye disorders coexisting with multiple sclerosis. Furthermore, pharmaceutical agents traditionally employed for allograft rejection exhibited promising therapeutic potential for the eye disorders coexisting with multiple sclerosis.

**Discussion:**

Multiple sclerosis genetically contributes to the development of optic neuritis while mitigating the concurrent occurrence of cataract and macular degeneration. Further research is needed to validate these findings and explore additional mechanisms underlying the comorbidity of multiple sclerosis and eye disorders.

## 1 Introduction

Multiple sclerosis (MS) is an autoimmune disease characterized by inflammation, demyelination, and axonal loss in the central nervous system (CNS). Over the years, the prevalence and incidence rates of MS have been continuously increasing in both developed and developing countries (1, 2). Ocular pathology is a common occurrence in up to 80% of MS patients throughout the course of their disease (3). Among which, acute optic neuritis affects approximately 30% to 70% of individuals with MS at some point (4). Unfortunately, like MS, there is no specific cure for ocular pathology coexisting with MS. For optic neuritis coexisting with MS, the most commonly used corticotherapy may accelerate the visual recovery, but it has no long-term visual benefit (5). In addition, it is not the best treatment option for other complications of MS (6). Therefore, it is important to target prevention and new therapy of ocular pathology coexisting with MS by means of establishing causal links.

Furthermore, studies utilizing the 25-Item National Eye Institute Visual Functioning Questionnaire and a 10-Item Neuro-Ophthalmic Supplement have identified additional eye-related symptoms in MS patients, including decreased visual acuity, contrast sensitivity, binocular vision defects, visual field abnormalities, reduced color vision, blurred vision, and diplopia (3). The presence of these clinical symptoms raises questions about the potential involvement of other eye disorders in the progression of MS, aside from optic neuritis. In clinical practice, decreased visual acuity and blurred vision are common symptoms among patients with eye disorders (7). These disorders encompass refractive errors (myopia, hyperopia, and astigmatism), macular degeneration (dry and wet), glaucoma, and cataracts (senile and other types). However, the causal relationship between these eye disorders and MS still remains elusive.

Mendelian randomization (MR) analysis, which follows the same design principles as a randomized controlled trial (RCT), utilizes single-nucleotide polymorphisms (SNPs) as instrumental variables (IVs) to discover a causal relationship between exposures and outcomes (8). Given the clinical coexistence of MS and eye disorders, we employ MR analysis to examine the genetic relationships between these two conditions. Furthermore, the extracted causal SNPs were annotated and the targeted gene expression was identified using the expression quantitative trait loci (eQTL) database. The biological function analysis of the targeted genes revealed that lymphocyte proliferation, activation and antigen processing might be involved in the pathogenesis of eye disorders coexisting with MS. Additionally, we also predicted that the drugs used for allograft rejection could potentially serve as a viable therapeutic intervention for patients simultaneously suffering from ocular disorders and MS. Overall, our study provides a new insight into the pathogenesis of MS coexisting with eye disorders.

## 2 Materials and methods

### 2.1 GWAS summary statistic data

**Figure 1:**
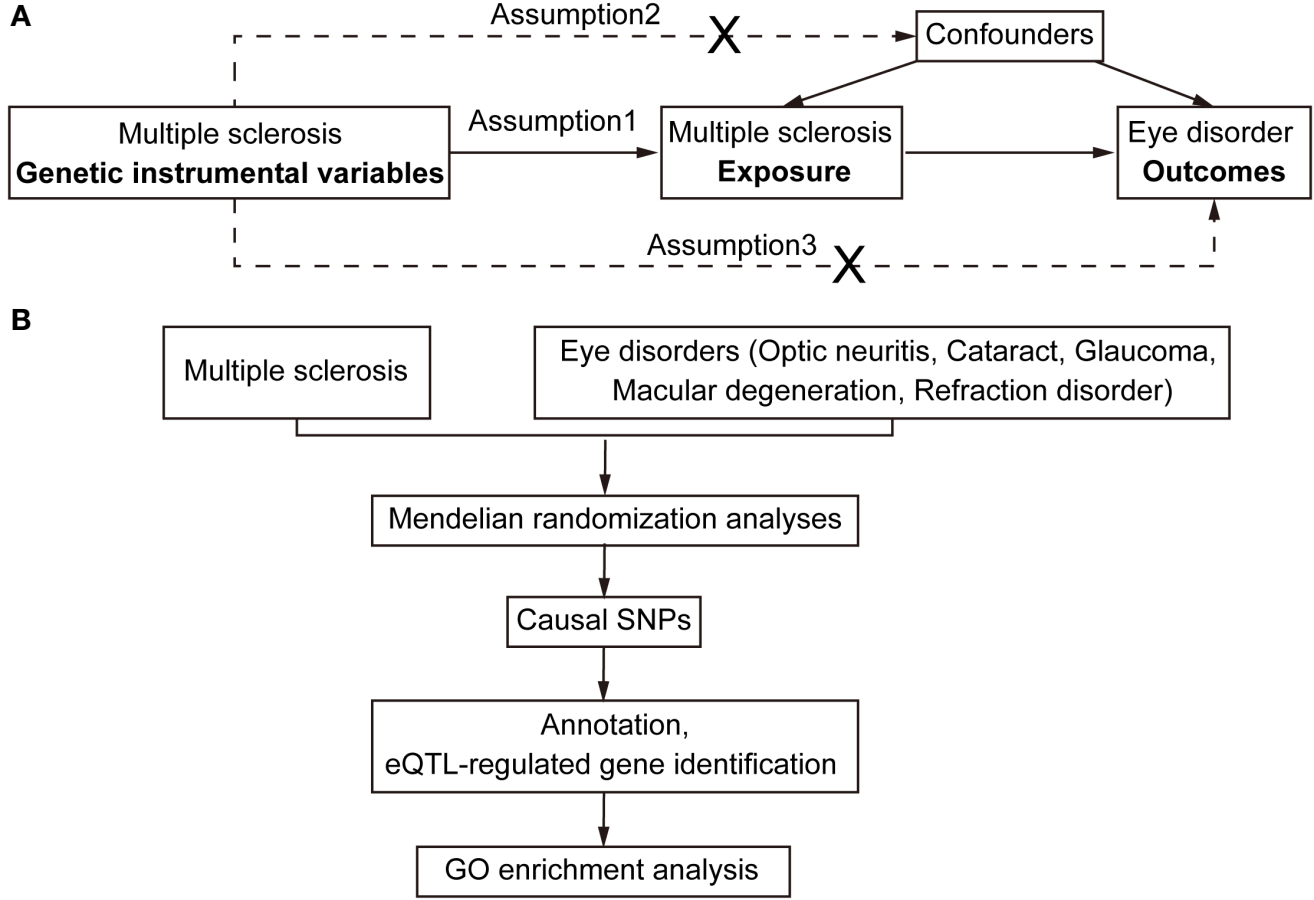
Flowchart for Mendelian randomization analysis. **(A)** Directed acyclic graph representing the Mendelian randomization framework for investigating the causal relationship between MS and eye disorders. Assumptions for instrumental variables: (1) The instruments must be associated with the exposures; (2) The instruments must influence eye disorders only through their impact on MS; and (3) The instruments must remain independent of both observed and unobserved confounding factors affecting the relationship between MS and eye disorders. **(B)** The work-flow of this study. Summary data of GWAS traits were extracted for detecting causal relationships between MS and eye disorders (optic neuritis, cataract, glaucoma, macular degeneration and refraction disorder). Subsequently, the causal SNPs were then annotated and mapped to the plasma eQTL. Finally, the identified putatively causal genes were used for enrichment analysis.

We investigated the genetic causality between MS and five commonly occurring eye disorders that might be misdiagnosed in the early phase of MS, namely optic neuritis, glaucoma, cataract, refraction disorder and macular degeneration. Although the symptoms of these eye disorders are all related to vision loss, the etiological factors vary among them. Optic neuritis is caused by the immune system that mistakenly targets the optic nerve, resulting in optic nerve inflammation. Glaucoma is a group of eye conditions (raised intraocular pressure, inflammation etc.) that damage the optic nerve. Cataract is a clouding of the lens, which can be caused by increasing aging (senile cataract), eye injury and inflammation etc. (other cataract). Refraction disorder occurs when the shape alteration of the lens prevents light from focusing directly on the retina. There are two types of macular degeneration. Dry age-related macular degeneration is due to the breaking down of the inner layers of the macula, and the wet age-related macular degeneration is caused by blood vessels that leak fluid or blood into the macula. The comprehensive design of the current study is depicted in **Figure 1**. We obtained GWAS summary statistic data from the GWAS catalog (https://gwas.mrcieu.ac.uk/). Four MS traits, including finn-b-G6_MS, ukb-b-17670, ebi-a-GCST005531 and ieu-a-1025 (9, 10), were selected to validate the results. For the eye disorders, we utilized the most recent traits with European ancestry and the largest sample size available. **Table 1** provides detailed information about these traits. Specifically, the traits of eye disorders contain finn-b-H7_OPTNEURITIS, finn-b-H7_GLAUCSECINFLAM, finn-b-H7_CATARACTSENILE, finn-b-H7_CATARACTOTHER, finn-b-H7_OCUMUSCLE, finn-b-DRY_AMD and finn-b-WET_AMD.

**Table 1:** Detailed information of the GWAS datasets explored and prioritized instruments used for the Mendelian randomization analysis.

GWAS ID	Exposure	Year	Sample size	NO. of SNPs	P value
finn-b-G6_MS	Multiple sclerosis	2021	1048 cases/217141 controls	16380460	5×10^-8^
ukb-b-17670	Multiple sclerosis	2018	1679 cases/461251 controls	9851867	5×10^-8^
ebi-a-GCST005531	Multiple sclerosis	2013	14498 cases/24091 controls	132089	5×10^-8^
ieu-a-1025	Multiple sclerosis	2013	14498 cases/24091 controls	156632	5×10^-8^
finn-b-H7_OPTNEURITIS	Optic neuritis	2021	582 cases/217491 controls	16380463	5×10^-6^
finn-b-H7_GLAUCSECINFLAM	Glaucoma secondary to eye inflammation	2021	96 cases/210201 controls	16380446	5×10^-6^
finn-b-H7_CATARACTSENILE	Senile cataract	2021	26758 cases/189604 controls	16380461	5×10^-6^
finn-b-H7_CATARACTOTHER	Other cataract	2021	7873 cases/189604 controls	16380403	5×10^-6^
finn-b-H7_OCUMUSCLE	Refraction disorder	2021	7861cases/210931 controls	16380466	5×10^-6^
finn-b-DRY_AMD	Dry age-related macular degeneration	2021	2469 cases/206221 controls	16380423	5×10^-6^
finn-b-WET_AMD	Wet age-related macular degeneration	2021	2114 cases/206601 controls	16380422	5×10^-6^

### 2.2 Selection of genetic instruments

The SNPs used as exposure IVs for MS were selected with a p value less than 5 × 10^-8^. However, in the eye disorder traits, the p-value cut-off was set as 5 × 10^-6^ since there was a lack of enough IVs for evaluation at p value of 5 × 10^-8^. In addition, the linkage disequilibrium in the selected SNPs was clumped with r^2^ <0.1 based on the 1000 Genomes Project. The F-statistics of each selected IV were calculated using the mRnd method (11). The IVs with F-statistics less than 10 were excluded to ensure the strength of instruments.

### 2.3 Two-sample MR analysis

Two-sample MR analyses were conducted using three models: inverse-variance weighted (IVW), MR-Egger (ME), and weighted median (WM). The palindromic SNPs with intermediate allele frequencies were excluded from the MR analysis. The odds ratio (OR) and corresponding 95% confidence interval (CI) were calculated to show the relationship between exposure variables and outcome variables. We also performed Cochran’s Q test to assess the horizontal heterogeneity. A Cochrane’s Q value in the MR–Egger regression and IVW tests was evaluated. Heterogeneity was considered statistically significant when the P value of the Q value (P_Q-value_) was less than 0.05. In addition, MR-Egger regression was used to evaluate the pleiotropic effects of IVs. All analyses were performed using the TwoSampleMR package (version 0.5.6) in R software (version 4.1.2).

### 2.4 Functional evaluation of causal SNPs

The basic information about the causal SNPs, including rsID, location, and effect allele, was extracted using the ‘mr_singlesnp’ function in the TwoSampleMR R package. To explore whether these causal SNPs affect gene expression, we analyzed eQTL-targeted genes in whole blood and identified motif alterations using the VannoPortal database (12). Additionally, we conducted Gene Ontology (GO) enrichment analysis and network cluster analysis of targeted genes of eQTL using STRING to elucidate the affected biological processes. Furthermore, curated pathway was explored using Cytoscape to identify potential therapeutic targets and pharmaceutical agents for the eye disorders coexisting with multiple sclerosis.

## 3 Results

### 3.1 The presence of MS facilitates optic neuritis pathology

**Figure 2:**
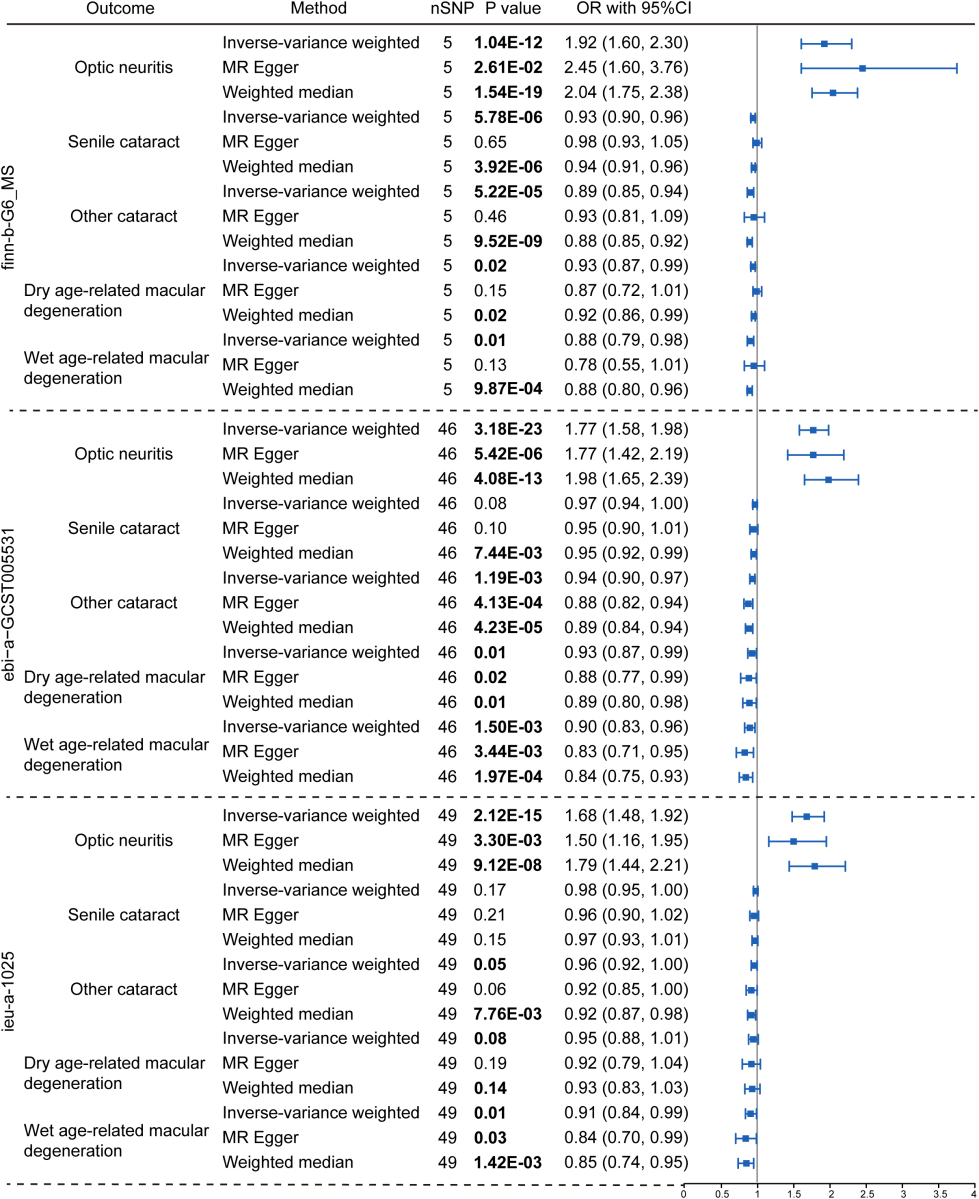
Causal relationship between multiple sclerosis and the risk of eye disorders. Data were presented as OR with 95% CI. Three GWAS traits of multiple sclerosis, including finn-b-G6_MS, ebi-a-GCST005531 and ieu-a-1025, were utilized as exposure variables respectively. Optic neuritis, senile cataract, other cataract, dry age-related macular degeneration and wet age-related macular degeneration were considered as outcomes. Three Mendelian randomization analysis methods were applied: the IVW, MR Egger and weighted median method. The nSNP refers to the number of SNP.

**Figure 3:**
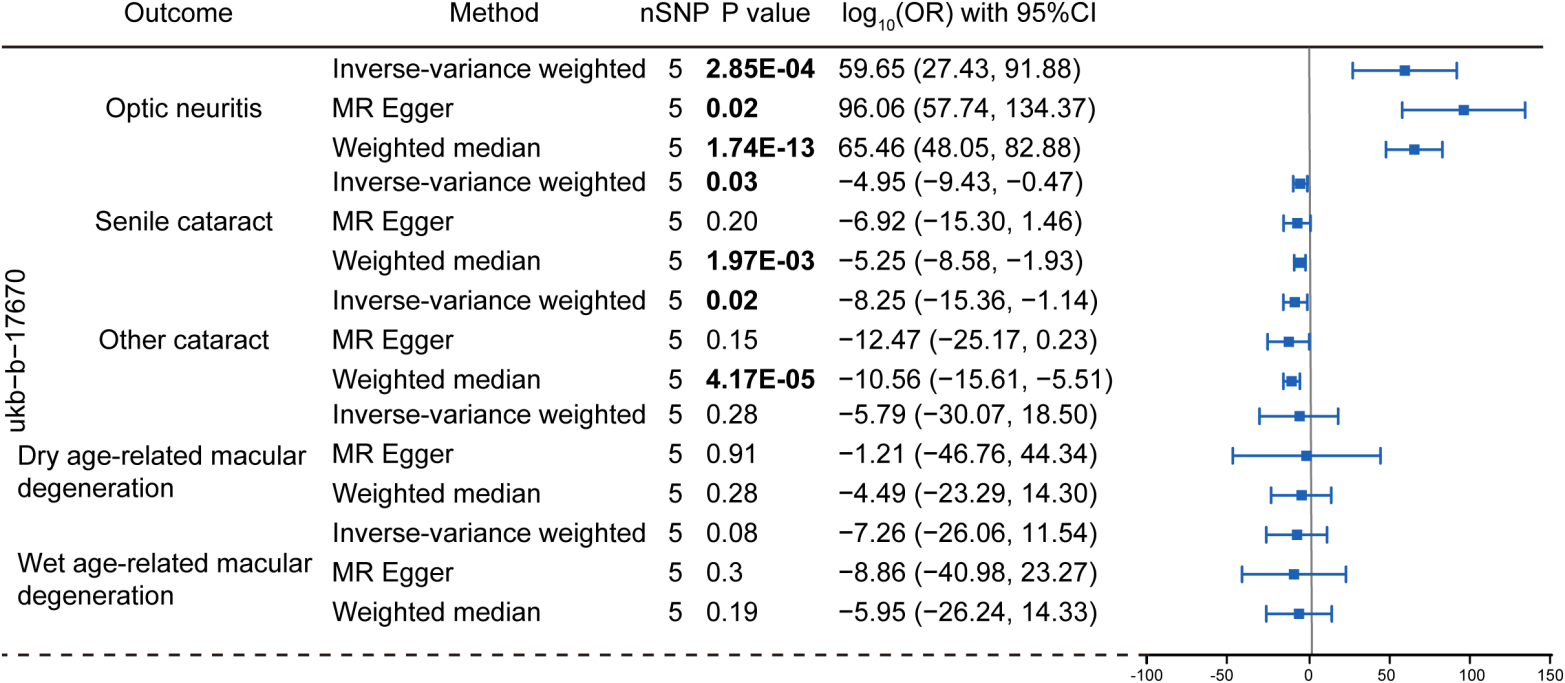
Mendelian randomization analysis assessing the causal link between multiple sclerosis (dataset: ubk-b-17670) and the risk of eye disorders. Data were presented as log-transformed odds ratios (log-OR) along with 95% CI. The genetic traits associated with multiple sclerosis from the ukb-b-17670 were employed as the exposure of interest. The considered eye disorders encompass optic neuritis, senile cataract, other cataract, dry age-related macular degeneration, and wet age-related macular degeneration as the outcome variables. Three distinct models, including the IVW, MR Egger and weighted median models were utilized for Mendelian randomization analysis. The nSNP refers to the number of SNP.

To explore the genetic relationship between MS and optic neuritis, a two-sample MR was performed. Four different MS datasets (GWAS ID: finn-b-G6_MS, ukb-b-17670, ebi-a-GCST005531, ieu-a-1025) and one optic neuritis dataset (GWAS ID: finn-b-H7_OPTNEURITIS) were selected (**Table 1**). The MR results from the IVW method showed that MS was causally associated with a higher risk of optic neuritis (finn-b-G6_MS: OR = 1.92, 95% CI = 1.60 - 2.30, P = 1.04× 10^-12^; ukb-b-17670: OR = 59.65, 95% CI = 27.43 - 91.88, P = 2.85× 10^-4^; ebi-a-GCST005531: OR= 1.77, 95% CI = 1.58 - 1.98, P = 3.18× 10^-23^; ieu-a-1025: OR = 1.68, 95% CI = 1.48 - 1.92, P = 2.12× 10^-15^, **Figures 2**, **3**). Considering potential horizontal heterogeneity or pleiotropy in the SNP effects (**Table 2**), the MR-Egger and Weighted median methods were used to validate the causal effect. The results confirmed the robust and significant causal relationship between MS and optic neuritis (**Figures 2**, **3**).

**Table 2:** Heterogeneity and pleiotropy tests of the Mendelian randomization analysis between MS and eye disorders.

Exposure	Outcome	Q value		P_Q-value_		Intercept	P_intercept_
		MR Egger	Inverse- variance weighted	MR Egger	Inverse- variance weighted		
finn-b-G6_MS	Optic neuritis	6.16	9.22	0.10	0.06	-0.21	0.31
	Senile cataract	3.47	7.86	0.32	0.10	-0.05	0.15
	Other cataract	9.39	10.82	0.02	0.03	-0.04	0.55
	Dry age-related macular degeneration	0.82	1.96	0.84	0.74	0.07	0.36
	Wet age-related macular degeneration	6.31	9.11	0.10	0.06	0.11	0.33
ebi-a- GCST005531	Optic neuritis	42.58	42.58	0.53	0.58	5.81E-04	0.98
	Senile cataract	87.48	88.95	1.05E-03	1.03E-03	4.47E-03	0.39
	Other cataract	53.35	60.05	0.16	0.07	0.01	0.02
	Dry age-related macular degeneration	40.46	41.91	0.62	0.60	0.01	0.24
	Wet age-related macular degeneration	40.76	49.46	0.36	0.30	0.02	0.12
ieu-a-1025	Optic neuritis	50.96	52.03	0.32	0.32	0.02	0.33
	Senile cataract	80.08	80.92	1.86E-03	2.07E-03	3.68E-03	0.49
	Other cataract	61.15	62.74	0.08	0.07	7.63E-03	0.27
	Dry age-related macular degeneration	41.04	41.32	0.72	0.74	0.01	0.60
	Wet age-related macular degeneration	53.89	55.67	0.23	0.21	0.02	0.22
ukb-b-17670	Optic neuritis	6.64	18.30	0.08	1.08E-03	-0.18	0.11
	Senile cataract	8.66	9.60	0.03	0.05	9.66E-03	0.61
	Other cataract	8.81	10.70	0.03	0.03	0.02	0.48
	Dry age-related macular degeneration	7.05	7.79	0.07	0.10	-0.02	0.62
	Wet age-related macular degeneration	3.01	3.08	0.39	0.54	0.01	0.80

**Figure 4:**
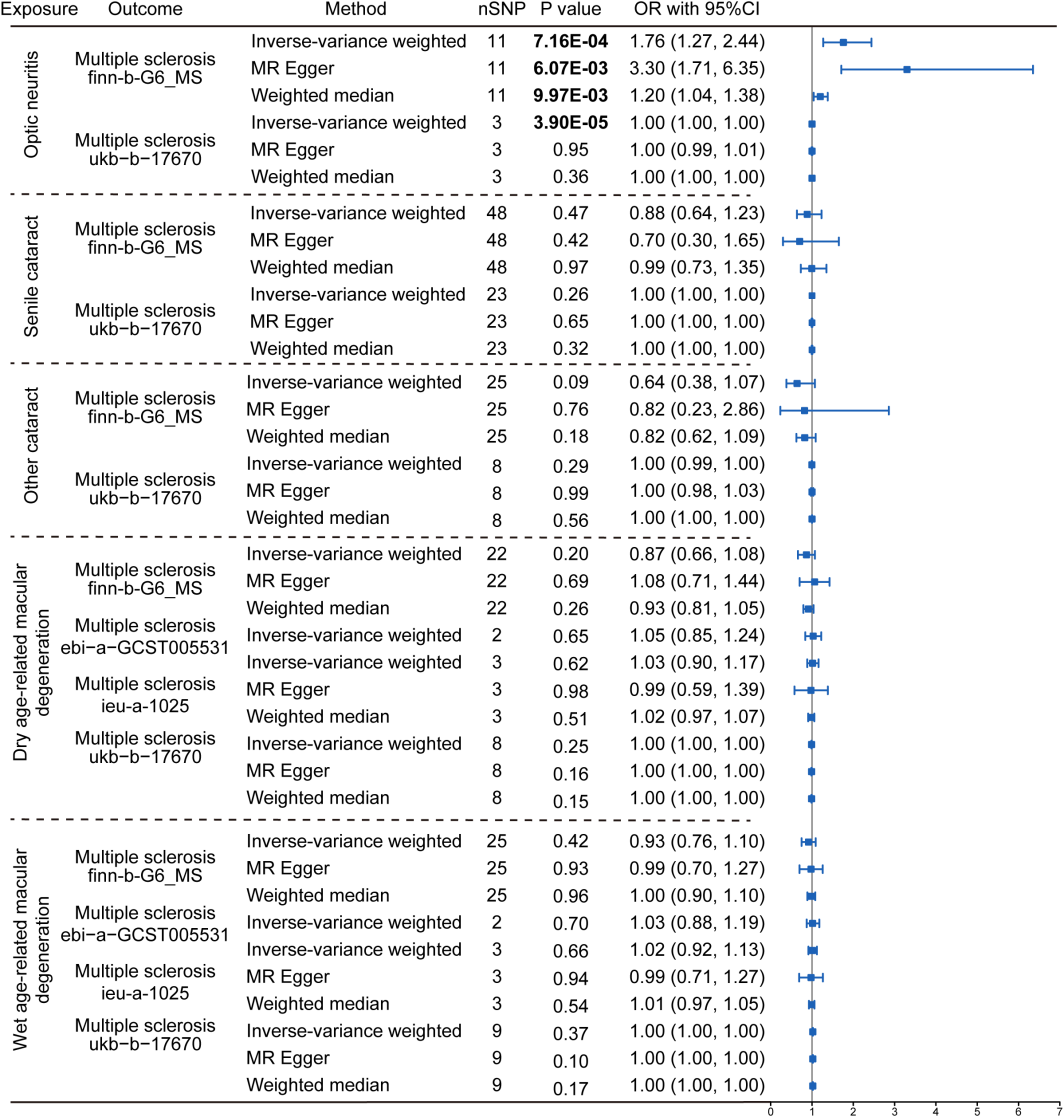
Reverse Mendelian randomization estimating the causal relationship between eye disorders and multiple sclerosis. The data are expressed in the form of OR accompanied by 95% CI. The different eye disorders served as the exposure variables, while MS was the designated outcomes. Only datasets for MS that contained a sufficient number of SNPs were included in the analysis. Three distinct Mendelian Randomization models were employed, including the IVW method, MR Egger, and the Weighted Median method. The nSNP refers to the number of SNP.

The reversal MR analyses (IVW method) showed that the causal link between optic neuritis and MS was only observed in the two selected datasets (finn-b-G6_MS: OR = 1.76, 95% CI = 1.27–2.44, *P* = 7.16× 10^-4^; ukb-b-17670: OR = 1.00, 95% CI = 1.00–1.00, *P* = 3.90× 10^-5^, **Figure 4**). The outcome datasets ebi-a-GCST005531 and ieu-a-1025 lacked an adequate number of IVs to conduct a MR analysis, indicating that optic neuritis is not strongly associated with the risk of MS. Collectively, the presence of MS is postulated to potentially increase the risk of optic neuritis but not vice versa. This finding is consistent with the widely accepted notion that optic neuritis is one of the most common complications of MS in clinics, and provide the genetic information to support the theory.

### 3.2 MS is genetically associated with a decreased risk of non-senile cataract wet age-related macular degeneration

We then explored the causal relationship between MS and other eye disorders, including cataract, macular degeneration, glaucoma and refraction disorder, since patients with these diseases also having similar vision problems as MS patients. There was a lack of genetic causality between MS and senile cataract and dry macular degeneration (**Figures 2**, **3**). However, the MR analysis (IVW method) revealed that MS was genetically related to a lower risk of other type of cataract (finn-b-G6_MS, OR = 0.89, 95% CI = 0.85–0.94, *P* = 5.22× 10^-5^; ukb-b-17670, OR = -8.25, 95% CI = -15.36–1.14, *P* = 0.02; ebi-a-GCST005531, OR= 0.94, 95% CI = 0.90–0.97, *P* = 1.19× 10^-3^; ieu-a-1025, OR = 0.96, 95% CI = 0.92–1.00, *P* = 0.05, **Figures 2**, **3**). The similar results were found using the MR-Egger (ebi-a-GCST005531: OR= 0.88, 95% CI = 0.82–0.94, *P* = 4.13× 10^-4^, **Figures 2**, **3**) and weighted median methods (finn-b-G6_MS: OR = 0.88, 95% CI = 0.85–0.92, *P* = 9.52× 10^-9^; ukb-b-17670: OR = -10.56, 95% CI = -15.61–5.51, *P* = 4.17× 10^-5^, **Figures 2**, **3**), indicating a stable and strong causality between MS and other type of cataract. In addition, causality between MS and wet age-related macular degeneration was found in three MS datasets using the IVW method (finn-b-G6_MS, OR = 0.88, 95% CI = 0.79 –0.98, *P* = 0.01; ebi-a-GCST005531, OR = 0.90, 95% CI = 0.83 –0.96, *P* = 1.5× 10^-3^; ieu-a-1025, OR = 0.91, 95% CI = 0.84 –0.99, *P* = 0.01) (**Figures 2**, **3**), indicating a weak causality between MS and wet age-related macular degeneration. However, we did not find any significant causality between MS and glaucoma or refraction disorder (**Supplementary Figure 1**), and vice versa (**Figure 4**; **Supplementary Figure 1**). In summary, our findings suggest that MS was negatively associated with other types of cataracts and wet age-related macular degeneration, but not with senile cataract, dry age-related macular degeneration, glaucoma, or refraction disorders.

### 3.3 The causal association between MS and eye disorders could be mediated by the lymphocyte proliferation, activation and antigen processing

**Figure 5:**
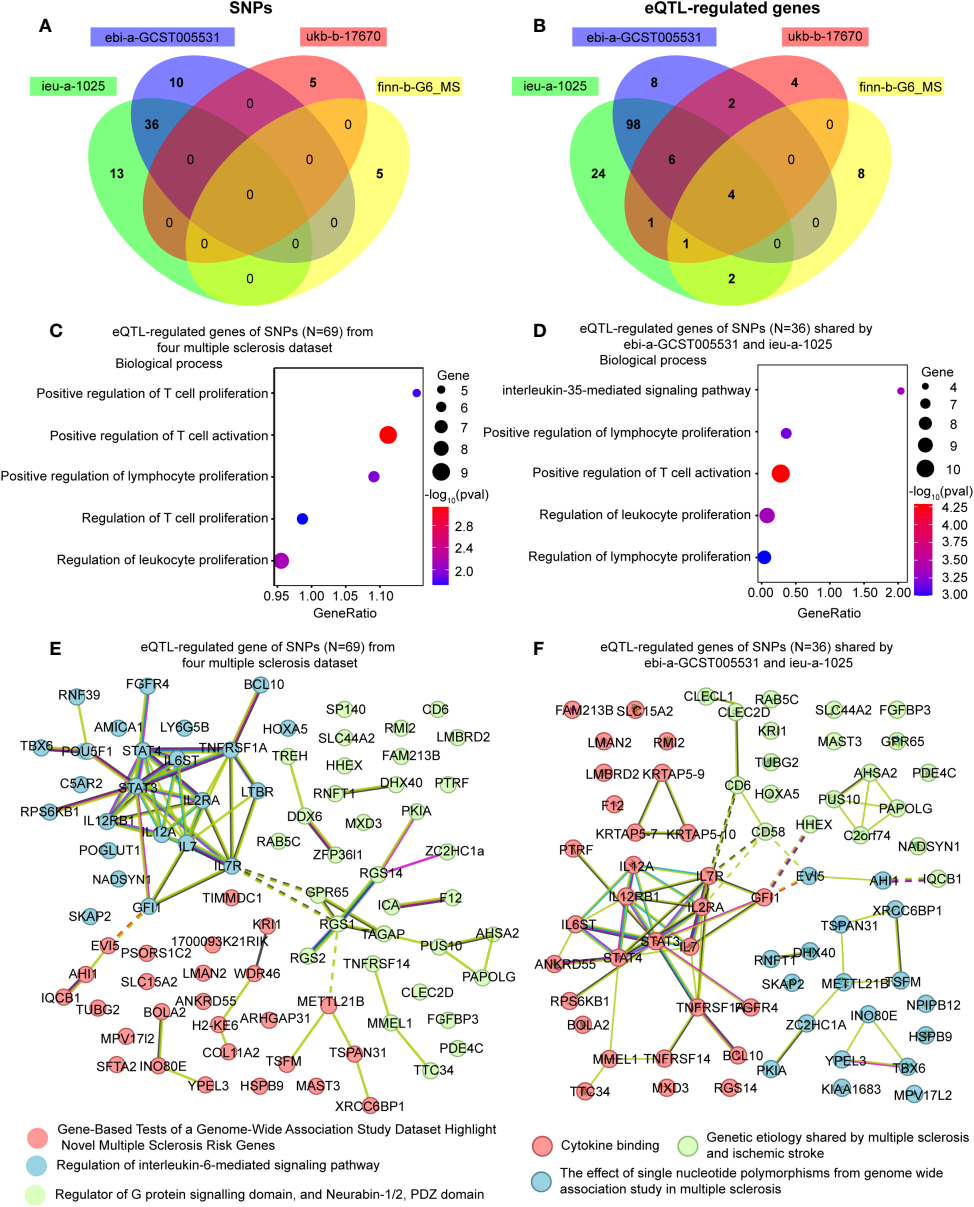
Enrichment analysis and the network of the eQTL-regulated genes from all causal SNPs. **(A)** A Venn diagram showing the shared SNPs among various datasets. **(B)** A Venn diagram illustrating the overlapping eQTL-regulated genes across distinct datasets. **(C)** Gene ontology enrichment analysis illustrating the biological processes enriched of eQTL-regulated genes from all causal SNPs (N = 69). **(D)** Gene ontology enrichment analysis illustrating the biological processes enriched of eQTL-regulated genes from the SNPs shared by ebi-a-GCST005531 and ieu-a-1025 (N = 36). **(E)** Network analysis of eQTL-regulated genes from all causal SNPs. **(F)** Network analysis of eQTL-regulated genes from the SNPs shared by ebi-a-GCST005531 and ieu-a-1025 (N = 36).

We collected a total of 69 causal SNPs from the exposure of four different MS datasets. Among them, 36 SNPs were shared by both ebi-a-GCST005531 and ieu-a-1025 datasets (**Figure 5A**). The causal SNPs were annotated using the VannoPortal Index database (**Table 3**), and it was found that the associated genes were related to JAK-STAT signaling pathway (IL12a, IL2Ra, IL20Ra, STAT3, STAT4, IL7R) and adaptive immunity (CD58, CD69, CD86, TNFSF14) by STRING clustering (Data not shown).

**Table 3:** eQTL-regulated genes of the causal SNPs from four MS datasets.

Exposure	SNP	Effect Allele	Nearest Gene	eQTL-regulated genes
finn-b-G6_MS	rs141298848	T	PDCD6IP	——
specific (n = 5)	rs149765808	A	ZSCAN12P1	——
	rs55782725	A	MICA	MICA, NCR3, PSORS1C1, C4A, PSORS1C2
	rs9264277	C	HLA-C	HCG27, XXbac-BPG181B23.7, PSORS1C3, MIR6891, HLA-L
	rs9271069	G	HLA-DRB1	HLA-DRB5, HLA-DRB6, HLA-DQB1, HLA-DQB1-AS1, HLA-DQA2
ukb-b-17670	rs28746956	G	HLA-DQB1	HLA-DQB2, HLA-DQB1, HLA-DQB1-AS1, HLA-DQA2, HLA-DQA
specific (n = 5)	rs4899257	T	ZFP36L1, RAD51B	CTD-2325P2.4, ZFP36L1
	rs67382147	G	HLA-DRB5	HLA-DRB5, HLA-DRB6, HLA-DQB1, HLA-DQB1-AS1, HLA-DRB1
	rs7759971	T	AHI1	AHI1, RP3-388E23.2, LINC00271
	rs9378141	C	MICD, HLA-W	HLA-J, IFITM4P, HLA-K, HCG4, MICD
ieu-a-1025	rs1131265	C	TIMMDC1	POGLUT1, ARHGAP31, TIMMDC1, RPL10P7
specific (n = 13)	rs115266049	A	TMPOP1, MICC	——
	rs115985474	T	TRIM31, RNF39	RPL23AP1, HLA-F, HCG4P7, IFITM4P, RNF39
	rs12210359	T	MCCD1P2, HCG4P3	HLA-J, HLA-K, HLA-A, MICD, SFTA2
	rs12296430	C	LTBR, RP1-102E24.1	RP1-102E24.8, LTBR
	rs1359062	G	RGS1, RGS21	RGS1, RP5-101101.2
	rs212405	T	TAGAP, FNDC1	RP1-111C20.4, TAGAP
	rs2857700	C	NCR3, UQCRHP1	HLA-DRB5, HLA-DRB6, HCG27, LY6G5B, HLA-C
	rs3129727	T	MTCO3P1, HLA-DQB3	HLA-DQB1-AS1, HLA-DQA2, HLA-DQB1, HLA-DRB6, HLA-DQB
	rs9263823	T	POU5F1	PSORS1C1, HLA-C, XXbac-BPG248L24.12, HCG22, POU5F1
	rs9277535	G	HLA-DPB1	HLA-DPB2, RPL32P1, HLA-DPA1, HSD17B8, HLA-DPB
	rs9736016	A	CXCR5, SETP16	DDX6, TREH, AP002954.4, JAML, TREHP1
	rs9989735	C	SP140	——
ebi-a-	rs1323292	A	RGS1, RGS21	RGS1, RP5-1011O1.2, RGS2
GCST005531	rs13426106	A	SP140	SP140, AC009950.2
specific (n = 10)	rs57271503	A	CD80	POGLUT1, ARHGAP31, TIMMDC1, RPL10P7
	rs3115627	G	MICF, HCG4P7	HLA-J, IFITM4P, HLA-K, HCG4, RPL23AP1
	rs9277641	A	HLA-DPB2	HLA-DPA1, COL11A2, HLA-DPB1, WDR46, HLA-DPB2
	rs4947337	C	C6orf10	——
	rs212407	A	TAGAP, FNDC1	RP1-111C20.4, TAGAP
	rs3130283	C	AGPAT1	HLA-DRB5, HLA-DRB6, HLA-DRB1, HLA-DQB1-AS1, HLA-DQB1
	rs10892299	T	CXCR5	——
	rs2364482	G	LTBR, RP1-102E24.1	LTBR, RP1-102E24.1
ebi-a-	rs1014486	C	IL12A	IL12A, IL12A-AS1
GCST005531	rs1021156	C	ZC2HC1A, PKIA	RP11-578O24.2, IL7, ZC2HC1A, PKIA, THAP12P7
and ieu-a-1025	rs10420809	T	SLC44A2	SLC44A2, KRI1
shared (n = 36)	rs1077667	T	TNFSF14	——
	rs11052877	G	CD69	CLECL1, RP11-705C15.2, DDX12P, CLEC2D, RP11-705C15.3
	rs11154801	A	AHI1	AHI1, RP3-388E23.2, LINC00271
	rs11172342	T	TSFM	METTL21B, TSFM, ATP23, TSPAN31, RP11-571M6.17
	rs11554159	A	IFI30, PIK3R2	MPV17L2, KIAA1683, PDE4C, MAST3, IL12RB1
	rs11865086	A	MAPK3	BOLA2, TBX6, NPIPB12, INO80E, YPEL3
	rs12087340	T	BCL10, DDAH1	BCL10
	rs12927355	T	CLEC16A	RP11-66H6.4, HNRNPCP4, RMI2
	rs17066096	G	IL20RA	——
	rs1800693	C	TNFRSF1A	TNFRSF1A
	rs1813375	T	EOMES	——
	rs2104286	C	IL2RA	IL2RA
ebi-a-	rs34383631	T	CD6	CD6
GCST005531	rs3748817	C	MMEL1	TTC34, MMEL1, FAM213B, TNFRSF14, RP3-395M20.8
and ieu-a-1025	rs41286801	T	EVI5	EVI5, GFI1
shared (n = 36)	rs4410871	C	MYC	PVT1
	rs4780355	C	RMI2	——
	rs4796791	C	STAT3	STAT3, PTRF, RAB5C, TUBG2, SPB9
	rs4944958	G	NADSYN1	RP11-660L16.2, NADSYN1, KRTAP5-7, KRTAP5-10, KRTAP5-9
	rs4976646	C	RGS14	RGS14, MXD3, FGFR4, LMAN2, F12
	rs60600003	G	ELMO1	——
	rs6677309	C	CD58	CD58, NAP1L4P1, RP5-1086K13.1
	rs67297943	C	TNFAIP3	——
	rs6881706	T	IL7R	LMBRD2, IL7R
	rs706015	G	SKAP2	HOTAIRM1, HOXA5, HOXA-AS3, HOXA-AS2, SKAP2
	rs71624119	A	ANKRD55	IL6ST, ANKRD55
	rs74796499	A	GALC	GPR65, LINC01146
	rs7783	G	CHERP, C19orf44	——
	rs7923837	A	EXOC6	EIF2S2P3, HHEX, TNKS2-AS1, FGFBP3
	rs8070345	C	VMP1	RNFT1, RP11-178C3.2, DHX40, DHX40P1, RPS6KB1
	rs842639	A	REL	C2orf74, PUS10, AHSA2, RP11-493E12.2, PAPOLG
	rs9282641	A	CD86	IQCB1, SLC15A2
	rs9967792	C	STAT4	STAT4

To further clarify the function of the causal SNPs, we searched for eQTL in whole blood (**Table 3**). A Venn diagram was used to visualize the shared eQTL genes in these four datasets, revealing four human leukocyte antigen (HLA) genes, including HLA-DRB5, HLA-DQB1-AS1, HLA-DRB6 and HLA-DQB1 (**Figure 5B**). GO enrichment analyses showed that lymphocyte proliferation and activation were enriched (**Figure 5C**). Additionally, the interleukin-35-mediated signaling pathway was enriched based on the genes associated with the shared SNPs between ebi-a-GCST005531 and ieu-a-1025 datasets (**Figure 5D**). Using the remaining SNPs (except for the shared ones), the interferon-gamma-mediated signaling pathway was identified (**Supplementary Figure 3**). STRING Protein-Protein interaction networks functional enrichment analysis showed that all the eQTL associated proteins were categorized into three clusters, mainly focused on MS risk genes, IL-6-mediated signaling pathway and the G-protein signaling domain (**Figure 5E**). The shared eQTL associated proteins were also categorized into three clusters, mainly enriched in cytokine signaling pathway (**Figure 5F**).

### 3.4 Potential therapeutic chemicals for MS coexisting with eye disorders

**Figure 6:**
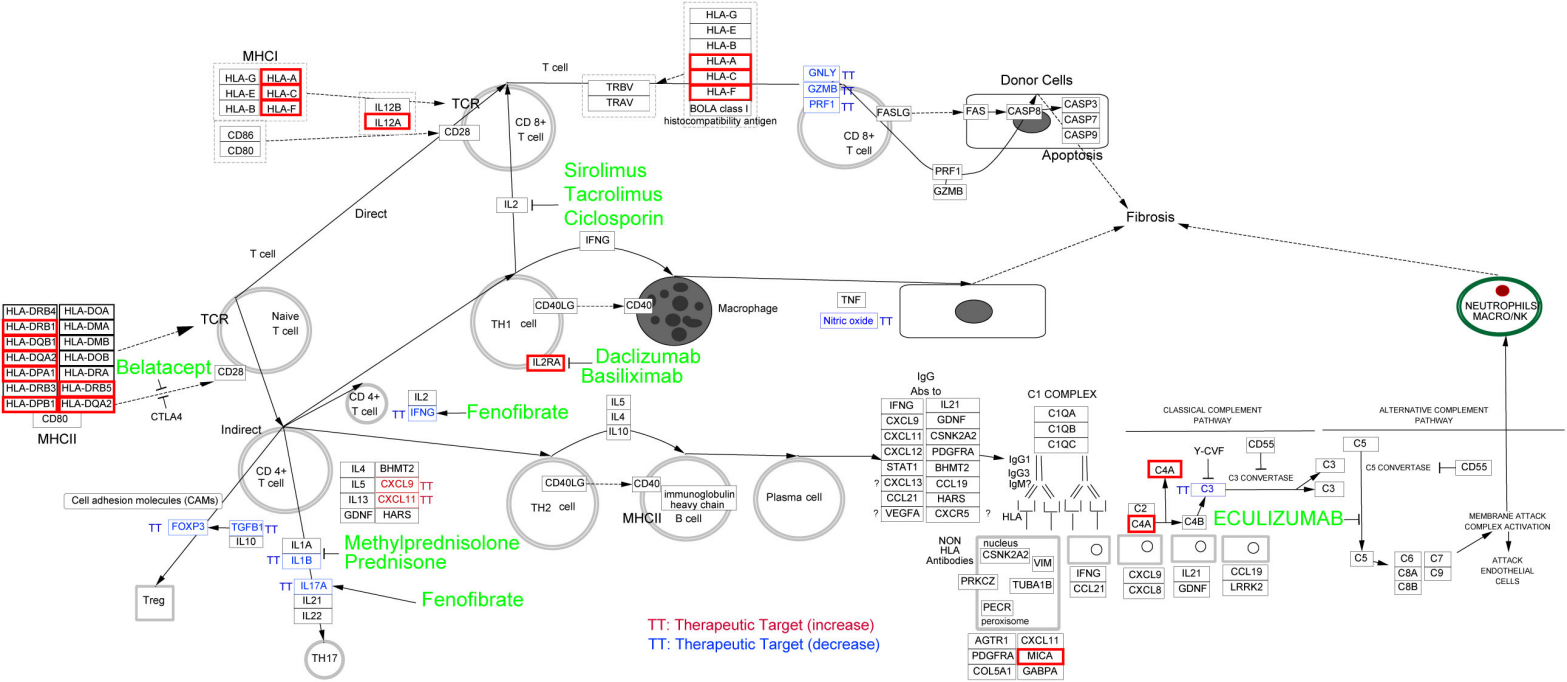
Potential drug discovering for MS coexisting with eye Disorders based on eQTL-regulated genes. Curated pathway analysis revealed that “allograft rejection” pathway was enriched. The associated eQTL-regulated genes were indicated by red boxes, and potential drugs to target related pathway were highlighted in green.

All the eQTL-regulated genes were used to explore the curated pathway using Cytoscope. Genes were mainly enriched in the “allograft rejection” pathway. Additionally, ten clinically used drugs were identified as inhibitors of this biological process. These chemicals targeted cytokine-mediated lymphocyte activation (sirolimus, tacrolimus, and ciclosporin for IL2-induced CD8^+^ T cell activation; Daclizumab and basiliximab for IL2RA-induced Th1 cell activation; Fenofibrate for IFEN-induced CD4^+^ T cell activation; Methylprednisolone, prednisone, and fenofibrate for Th17 cell activation), antigen processing (belatacept), and the complement system (eculizumab). A detailed list of the information and targets of these chemicals was shown in **Figure 6**.

## 4 Discussion

In this study, we analyzed the genetic relationships between MS and common eye disorders, such as optic neuritis, cataract, glaucoma, macular degeneration and refractive disorders, using MR analysis. Our data have revealed that (1) MS is genetically associated with optic neuritis pathology, but there is no evidence of a reciprocal relationship; (2) MS is causally related to reducing cataract and macular degeneration; (3) MS might influence the pathology of eye disorders through regulating the lymphocyte activation, proliferation and differentiation; (4) Drugs used for allograft rejection exhibit potential therapeutic effects on the MS coexisting with eye disorders. Our study provides genetic evidence regarding the incidence of and potential therapeutic targets for MS in conjunction with eye disorders.

### 4.1 MS and eye disorders

It has been widely accepted in clinics that optic neuritis, as an inflammatory demyelinating disorder of the optic nerve, can occur as a symptom of MS (13, 14). According to the North American Optic Neuritis Treatment Trial (ONTT), the risk of developing MS after 15 years was higher in patients who had abnormal brain scans than in those who had normal brain scans (15). However, patients with optic neuritis who lived in areas with low MS prevalence had lower rates of brain MRI abnormalities (14), indicating that optic neuritis might not be an independent risk factor for MS. It was supported that optic neuritis, in isolation, does not inherently involve the widespread autoimmune response observed in MS (16). Consistently, our investigation revealed a relatively limited causal relationship between optic neuritis and MS. However, the MR results showed that MS was a causal risk factor for optic neuritis. MS is a chronic inflammatory, demyelinating and neurodegenerative disease that affects the central nervous system (CNS). The demyelinating lesions in the optic nerve can impair the optic nerve in MS patients, leading to optic neuritis and even visual dysfunction (1).

We also identified that MS was causally related to reducing the incidence of other cataract and wet age-related macular degeneration. However, a population-based cohort study conducted using the UK General Practice Research Database from 1987 to 2009 reported that MS is not associated with the overall incidence of cataract or macular degeneration, but the risk of cataract is higher in MS patients younger than 50 years old, especially for men (17). MS and cataract share some mechanisms of disease pathological processes (17). The activity of proteolytic enzyme is markedly elevated in relapsing MS patients (18), and over-activation of proteolytic enzyme is thought to cause the degradation of lens proteins and changes in the cytoskeletal architecture, resulting in the formation of cataract (19). Additionally, prolonged exposure to corticosteroids, anticonvulsant and statin, commonly used in the treatment of MS, have been postulated as a potential risk factor for cataract development (20–23).

Some studies suggest an increased prevalence of age-related macular degeneration among patients with MS (24, 25). There is a potential interplay between the neurodegenerative processes occurring in MS and the retinal degeneration seen in age-related macular degeneration. Both conditions involve neuronal damage and degeneration, albeit in different anatomical locations (26). We speculate that the shared disease pathological processes, medication use and aging-associated diseases in patients might induce the discordance between clinical findings and our MR results.

### 4.2 MS and adaptive immune response

We have identified the causal relationship between MS and optic neuritis, cataract and macular degeneration using MR analysis. However, it still remains unclear how MS might influence the onset of eye disorders. After MS occurs, the adaptive immune system, especially T cells (Such as Th1 and Th17 cell) and B cells, becomes hyperactive and produces high levels of pro-inflammatory cytokines (27). In the present study, the enrichment analysis of targeted eQTL of extracted SNPs, revealed that the adaptive immune response, including T lymphocyte proliferation, activation, antigen processing and cytokine-mediated signaling was involved in the pathogenesis of MS coexisting with eye disorders. It is consistent with a previous report that adaptive immune cells infiltrated into the parenchyma, perivascular spaces, and meninges in optic nerve tissues in MS patients, even without visual disturbances (28). In the experimental autoimmune encephalomyelitis (EAE) model, the transcription of Th1 and Th17-specific cytokine genes interferon gamma (*Ifng) and* interleukin 17A (*Il17a*) was upregulated in optic nerve, and decreasing *Ifng* expression had a protective effect on optic neuritis in EAE mice (29). Additionally, aqueous humor IFNγ levels also increased in patients with either aged cataract or age-related macular degeneration (30, 31), indicating the involvement of Th cells in the pathogenesis of these two diseases. The above evidences support our findings that lymphocyte-induced immune response might play a role in MS coexisting with the eye disorders.

We also annotated the nearest genes (total 80 genes) of the causal SNPs from four MS datasets. There are thirty-four genes that were directly associated with the immune response and inflammation, and thirty genes were not. Other sixteen genes are pseudogenes. The detailed biological functions of all genes were listed in the **Supplementary Table 2**. Then we conducted functional enrichment analysis of the genes that were indirectly associated with inflammatory response, and found that the main enriched biological processes included asymmetric cell division, inositol lipid-mediated signaling, response to epidermal growth factor, process utilizing autophagic mechanism, and phagocytosis (**Supplementary Table 3**). It is worth noting that the epidermal growth factor signaling pathway play important role in eye diseases through enhancing the anti-apoptotic and anti-inflammatory effects of corneal epithelial cells (32). Anticancer drugs which target to epidermal growth factor receptor can inhibit corneal reparation and induce ocular toxicity (33), indicating that MS may also affect the occurrence of optic neuritis through non-inflammatory ways.

In the thirty-four inflammation associated genes, we identified several HLA family genes involved in the MS coexisting with eye disorders. HLA family genes are associated with adaptive immune response and have been reported to be related to the genetic susceptibility within the immune system, leading to MS (34, 35). Treatments based on genetic information have proven to be a promising path for drug repurposing and drug development. By using Cytoscape, we enriched the drugs that used for allograft rejection, suggesting their potential as therapeutic options for MS patients with eye disorders.

There are ten allograft rejection drugs predicted in our study. Among these drugs, some have already been the first-line treatment for MS (methylprednisolone and prednisone) (36). Additionally, others, including eculizumab, sirolimus, ciclosporine, tacrolimus, fenofibrate and daclizumab, have also been tested in clinical trials for MS. Eculizumab, a humanized monoclonal antibody approved for the treatment of aquaporin-4-positive neuromyelitis optica spectrum disorders, has undergone investigation for its safety and efficacy in MS (37). However, its efficacy as a disease-modifying therapy for MS patients still remains inconclusive due to study limitations (37, 38). Sirolimus, as a type of rapamycin inhibiting the mTOR kinase, is currently approved for lymphangioleiomyomatosis therapy (39). Recent studies have explored its potential in treating MS, with promising results suggesting its consideration as a therapeutic option with minimal side effects (40, 41). Ciclosporine and tacrolimus, inhibitors of calcineurin (an enzyme involved in the transcription of IL-2 and T-cell activation), have exhibited potential therapeutic effects for MS (42, 43). The PPAR alpha agonists such as fenofibrate, have been observed to ameliorate the clinical symptoms of MS through inhibiting interleukin-4 and interferon-γ secretion (44). Daclizumab, a monoclonal antibody targeting the CD25 subunit of the interleukin-2 receptor, has shown efficacy in slowing the inflammatory process of MS (45). Moreover, Afief and colleagues have reported that belatacept acted as a promising MS drug candidate using integration of genomic variants and bioinformatic-based approach (46). These studies support our prediction that allograft rejection drugs are potential therapeutics for MS, even MS coexisting with eye disorders.

The present study has several limitations. First, MS is more prevalent in women than in men, with a female-to-male ratio of approximately 3:1 (47, 48). However, the GWAS summary statistic datasets used in this study include both sexes, which could not distinguish the differences between women and men. The different gender ratio in the datasets could affect the MR results and introduce bias in the evaluation. Second, the prevalence of MS varies across different regions and ethnicities (49). We only used the datasets from European populations, which may limit generalizability of our findings to other populations. Third, the drug prediction was based on the causal SNPs, which should be interpreted with caution, as they may not reflect the true causal variants or genes. Additional limitations in our study are as followings. The sample size of the five eye disorders traits is relatively small, so we have adopted a relaxed p value of 1E-6 to screen for more exposure IVs, but this could compromise the reliability of our analysis. We have focused on the exposure of MS traits, which have robust sample size to perform the analysis. In addition, performing drug prediction by only using causal SNPs would leave out some important therapies, such as the widely used dihydroorotate-dehydrogenase inhibitor (teriflunomide).

In conclusion, we analyzed the causal associations between MS and common eye disorders using MR to explore the mechanisms of MS coexisting with eye disorders. Our results reveal that MS is casually associated with a higher risk of optic neuritis and a lower risk of non-senile cataract and age-related macular degeneration. Bioinformatics analysis revealed that lymphocyte proliferation, activation and antigen processing are involved in the pathogenesis of MS coexisting with eye disorders. These findings provide a better understanding of the etiology of MS and potential therapeutic targets in MS patients with eye disorders.

## Data availability statement

The original contributions presented in the study are included in the article/**Supplementary Material**. Further inquiries can be directed to the corresponding authors.

## Author contributions

XQ: Data curation, Formal analysis, Funding acquisition, Investigation, Writing – review & editing. MNH: Funding acquisition, Supervision, Writing – review & editing. SP: Conceptualization, Funding acquisition, Validation, Writing – original draft.
