# Supplementary material for: Genetic susceptibility and causal pathway analysis of eye disorders coexisting in multiple sclerosis
Source: Front Immunol. 2024 Feb 5;15:1337528. doi: 10.3389/fimmu.2024.1337528 (PMC10875133; doi:10.3389/fimmu.2024.1337528)
Supplement: Supplementary file 1 [file DataSheet_1.docx]

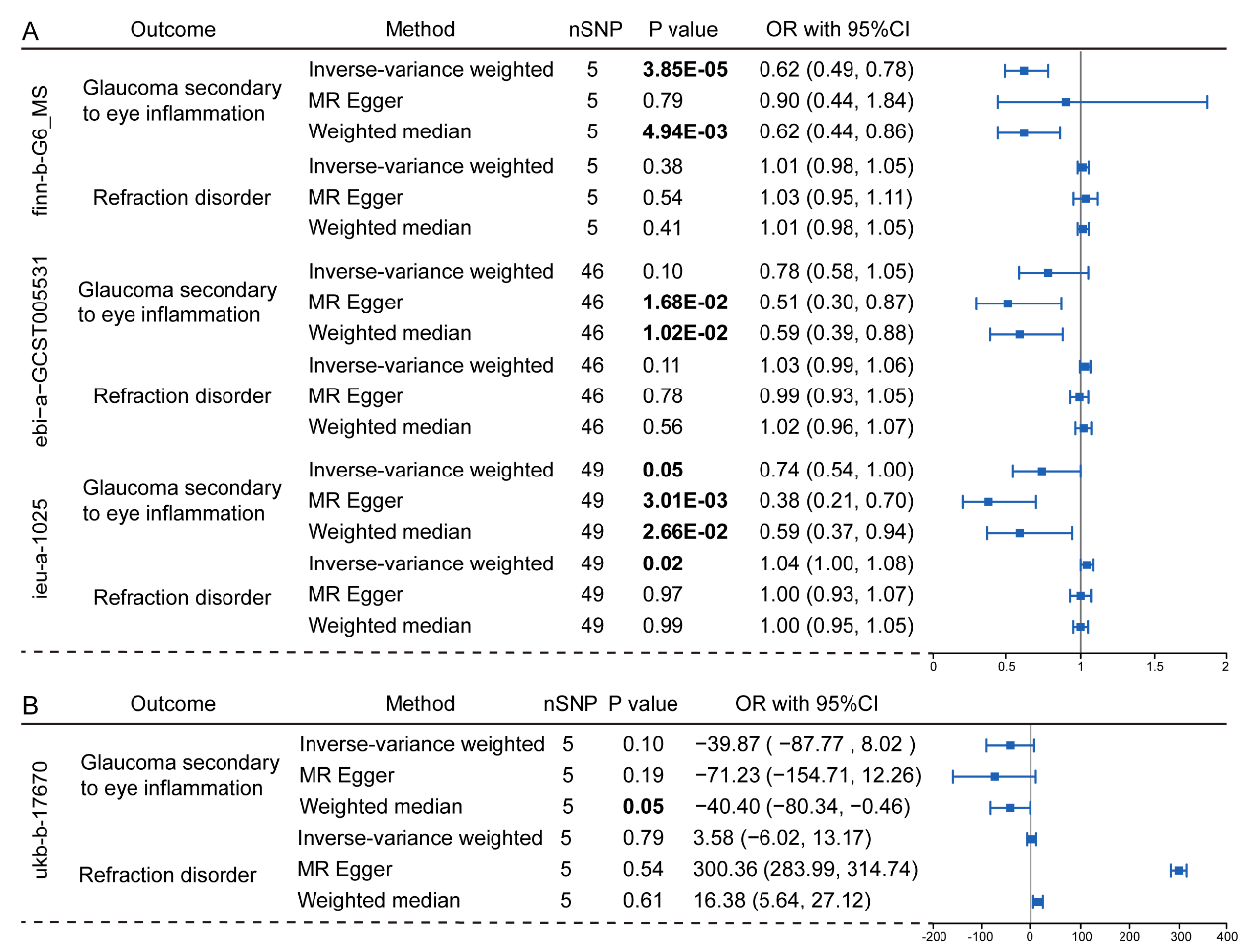
**Supplementary figure 1 Causal relationship between multiple sclerosis and glaucoma secondary to eye inflammation or refraction disorder.** Data were presented as OR with 95% CI. GWAS traits of exposure in the analysis included finn-b-G6_MS, ebi-a-GCST005531, ieu-a-1025 (A) and ukb-b-17670 (B). MR, Mendelian randomization; SNP, single nucleotide polymorphisms; nSNP, the number of SNP; IVW, inverse-variance weighted method.


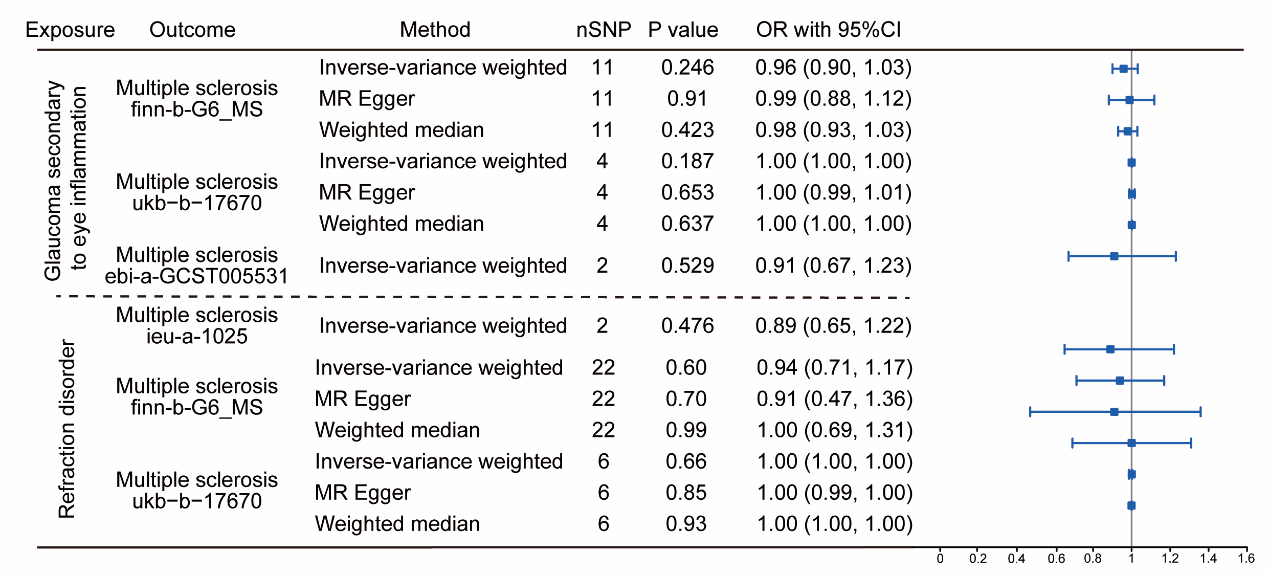
**Supplementary figure 2 Reverse Mendelian randomization estimates the causal association between eye disorders (glaucoma secondary to eye inflammation and refraction disorder) and the risk of MS.** Data were presented as OR with 95% CI. MR, Mendelian randomization; SNP, single nucleotide polymorphisms; nSNP, the number of SNP; IVW, inverse-variance weighted method.

**Supplementary figure 3 Enrichment analysis and the network of the eQTL-regulated genes from the shared causal SNPs.**
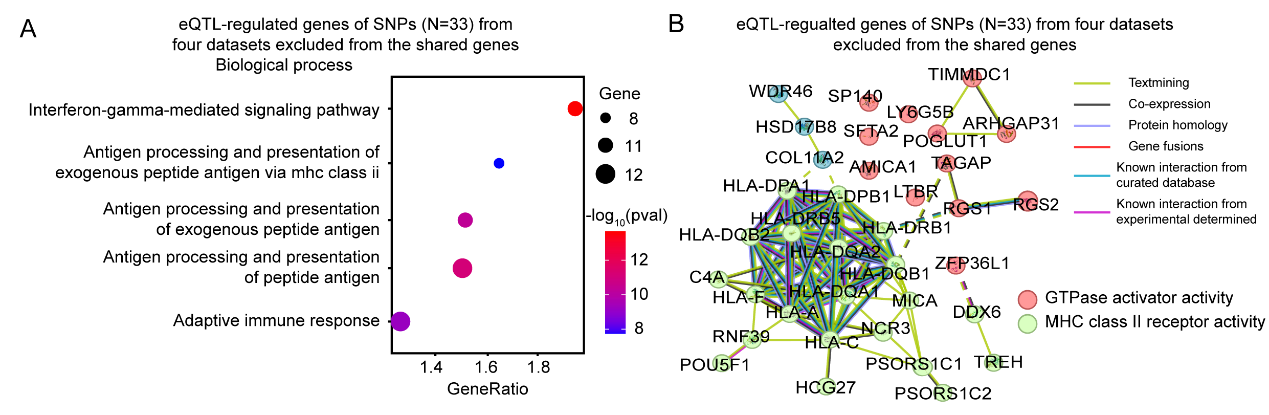
 (A) Biological processes enriched of eQTL-regulated genes from the shared causal SNPs (N = 33, shared by two datasets of MS exposure: ebi-a-GCST005531 and ieu-a-1025). (B) Network analysis displaying the interconnections among eQTL-regulated genes derived from the shared causal SNPs.
