# Supplementary material for: Genetic susceptibility and causal pathway analysis of eye disorders coexisting in multiple sclerosis
Source: Front Immunol. 2024 Feb 5;15:1337528. doi: 10.3389/fimmu.2024.1337528 (PMC10875133; doi:10.3389/fimmu.2024.1337528)
Supplement: Supplementary file 2 [file Table_1.docx]

**Supplementary table1** Heterogeneity and pleiotropy tests of the Mendelian randomization analysis between MS and glaucoma secondary to eye inflammation, or refraction disorder.

| Exposure | Outcome | Q value | | P_Q-value_ | | Intercept | P_intercept_ |
| --- | --- | --- | --- | --- | --- | --- | --- |
|  |  | MR Egger | Inverse-variance  weighted | MR Egger | Inverse-variance  weighted |  |  |
| finn-b-G6_MS | Glaucoma secondary to eye inflammation | 1.42 | 2.66 | 0.70 | 0.62 | -0.33 | 0.35 |
|  | Refraction disorder | 0.23 | 0.37 | 0.97 | 0.99 | -0.01 | 0.18 |
| ebi-a-  GCST005531 | Glaucoma secondary to eye inflammation | 45.47 | 49.06 | 0.41 | 0.31 | 0.09 | 0.18 |
|  | Refraction disorder | 49.73 | 51.87 | 0.26 | 0.22 | 0.01 | 0.02 |
| ieu-a-1025 | Glaucoma secondary to eye inflammation | 43.71 | 49.80 | 0.61 | 0.40 | 0.12 | 0.02 |
|  | Refraction disorder | 49.08 | 50.98 | 0.39 | 0.36 | 0.01 | 0.18 |
| ukb-b-17670 | Glaucoma secondary to eye inflammation | 5.45 | 6.95 | 0.14 | 0.14 | 0.15 | 0.43 |
|  | Refraction disorder | 2.41 | 2.84 | 0.49 | 0.58 | -0.01 | 0.56 |
