# Supplementary material for: Genetic susceptibility and causal pathway analysis of eye disorders coexisting in multiple sclerosis
Source: Front Immunol. 2024 Feb 5;15:1337528. doi: 10.3389/fimmu.2024.1337528 (PMC10875133; doi:10.3389/fimmu.2024.1337528)
Supplement: Supplementary file 3 [file Table_2.docx]

**Supplementary table 2** Biological function of nearest genes of the causal SNPs from four MS datasets.

| **Nearest gene** | | **Biological function** |
| --- | --- | --- |
| Directly associated with the immune response and inflammation | BCL10 | Involved in adaptive immune response. |
|  | CD58 | Ligand of the T-lymphocyte CD2 glycoprotein. |
|  | CD6 | Cell adhesion molecule that mediates cell-cell contacts and regulates T-cell responses via its interaction with ALCAM/CD166. |
|  | CD69 | Involved in lymphocyte proliferation and functions as a signal transmitting receptor in lymphocytes, natural killer (NK) cells, and platelets. |
|  | CD80 | Involved in the costimulatory signal essential for T- lymphocyte activation. |
|  | CD86 | Involved in the costimulatory signal essential for T-lymphocyte proliferation and interleukin-2 production, by binding CD28 or CTLA-4. |
|  | CXCR5 | Cytokine receptor that binds to B-lymphocyte chemoattractant (BLC). |
|  | EOMES | Involved in the differentiation of CD8+ T-cells during immune response regulating the expression of lytic effector genes. |
|  | EVI5 | Functions as a regulator of cell cycle progression by stabilizing the FBXO5 protein and promoting cyclin-A accumulation during interphase. May play a role in cytokinesis. |
|  | HLA-C | Antigen-presenting MHCI molecule with an important role in reproduction and antiviral immunity. |
|  | HLA-DPB1 | Binds peptides derived from antigens that access the endocytic route of antigen presenting cells and presents them on the cell surface for recognition by the CD4 T-cells. |
|  | HLA-DQB1 | Binds peptides derived from antigens that access the endocytic route of antigen presenting cells and presents them on the cell surface for recognition by the CD4 T-cells. |
|  | HLA-DRB1 | A beta chain of antigen-presenting major histocompatibility complex class II (MHCII) molecule. |
|  | HLA-DRB5 | Binds peptides derived from antigens that access the endocytic route of antigen presenting cells and presents them on the cell surface for recognition by the CD4 T-cells. |
|  | IFI30 | Lysosomal thiol reductase that can reduce protein disulfide bonds.Plays an important role in antigen processing. |
|  | IL12A | Cytokine that can act as a growth factor for activated T and NK cells, enhance the lytic activity of NK/lymphokine-activated killer cells, and stimulate the production of IFN-gamma by resting PBMC |
|  | IL20RA | The IL20RA/IL20RB dimer is a receptor for IL19, IL20 and IL24. The IL20RA/IL10RB dimer is a receptor for IL26 |
|  | IL2RA | The receptor is involved in the regulation of immune tolerance by controlling regulatory T cells (TREGs) activity. TREGs suppress the activation and expansion of autoreactive T-cells. |

**Supplementary table 2 (continued)**

| Directly associated with the immune response and inflammation | IL7R | Receptor for interleukin-7. Also acts as a receptor for thymic stromal lymphopoietin (TSLP); Belongs to the type I cytokine receptor family. |
| --- | --- | --- |
|  | LTBR | Receptor for the heterotrimeric lymphotoxin containing LTA and LTB, and for TNFS14/LIGHT. |
|  | MICA | Acts as a stress-induced self-antigen that is recognized by gamma delta T-cells. Ligand for the KLRK1/NKG2D receptor. Binding to KLRK1 leads to cell lysis. Belongs to the MHC class I family. MIC subfamily. |
|  | MYC | Transcription factor that binds DNA in a non-specific manner, yet also specifically recognizes the core sequence 5'-CAC[GA]TG-3'. |
|  | NCR3 | Cell membrane receptor of natural killer/NK cells that is activated by binding of extracellular ligands including BAG6 and NCR3LG1. |
|  | REL | Proto-oncogene that may play a role in differentiation and lymphopoiesis. |
|  | RGS1 | Regulates G protein-coupled receptor signaling cascades, including signaling downstream of the N-formylpeptide chemoattractant receptors and leukotriene receptors. Inhibits B cell chemotaxis toward CXCL12. |
|  | SKAP2 | May be involved in B-cell and macrophage adhesion processes. |
|  | SP140 | Component of the nuclear body, also known as nuclear domain 10, PML oncogenic domain, and KR body. May be involved in the pathogenesis of acute promyelocytic leukemia and viral infection. |
|  | STAT3 | Signal transducer and transcription activator that mediates cellular responses to interleukins, KITLG/SCF, LEP and other growth factors. |
|  | STAT4 | Carries out a dual function: signal transduction and activation of transcription. Involved in IL12 signaling. |
|  | TAGAP | May function as a GTPase-activating protein and may play important roles during T-cell activation. |
|  | TAGAP | May function as a GTPase-activating protein and may play important roles during T-cell activation. |
|  | TNFAIP3 | Involved in immune and inflammatory responses signaled by cytokines, such as TNF-alpha and IL-1 beta, or pathogens via Toll-like receptors (TLRs) through terminating NF-kappa-B activity. |
|  | TNFRSF1A | Receptor for TNFSF2/TNF-alpha and homotrimeric TNFSF1/lymphotoxin-alpha. |
|  | TNFSF14 | Cytokine that binds to TNFRSF3/LTBR. Binding to the decoy receptor TNFRSF6B modulates its effects. |

**Supplementary table 2 (continued)**

| Indirectly associated with the immune response and inflammation | AHI1 | Involved in vesicle trafficking and required for ciliogenesis, formation of primary non-motile cilium, and recruitment of RAB8A to the basal body of primary cilium. |
| --- | --- | --- |
|  | CLEC16A | Regulator of mitophagy through the upstream regulation of the RNF41/NRDP1-PRKN pathway. |
|  | GALC | Hydrolyzes the galactose ester bonds of galactosylceramide, galactosylsphingosine, lactosylceramide, and monogalactosyldiglyceride. |
|  | MAPK3 | Serine/threonine kinase which acts as an essential component of the MAP kinase signal transduction pathway. |
|  | NADSYN1 | Catalyzes the ATP-dependent amidation of deamido-NAD to form NAD. |
|  | RMI2 | Essential component of the RMI complex, a complex that plays an important role in the processing of homologous recombination intermediates. |
|  | TIMMDC1 | Chaperone protein involved in the assembly of the mitochondrial NADH:ubiquinone oxidoreductase complex (complex I) |
|  | ZC2HC1A | Zinc finger C2HC-type containing 1A |
|  | AGPAT1 | Converts lysophosphatidic acid or LPA into phosphatidic acid or PA by incorporating an acyl moiety at the sn-2 position of the glycerol backbone. |
|  | ANKRD55 | Ankyrin repeat domain 55. |
|  | C19orf44 | Chromosome 19 open reading frame 44 |
|  | CHERP | Involved in calcium homeostasis, growth and proliferation. |
|  | DDAH1 | Hydrolyzes N(G), N(G)-dimethyl-L-arginine (ADMA) and N(G)- monomethyl-L-arginine (MMA) which act as inhibitors of NOS. |
|  | ELMO1 | Involved in cytoskeletal rearrangements required for phagocytosis of apoptotic cells and cell motility. |
|  | EXOC6 | Component of the exocyst complex involved in the docking of exocytic vesicles with fusion sites on the plasma membrane. |
|  | FNDC1 | May be an activator of G protein signaling |
|  | MMEL1 | Metalloprotease involved in sperm function, possibly by modulating the processes of fertilization and early embryonic development. |
|  | PDCD6IP | Multifunctional protein involved in endocytosis, multivesicular body biogenesis, membrane repair, cytokinesis, apoptosis and maintenance of tight junction integrity. |

**Supplementary table 2 (continued)**

| Indirectly associated with the immune response and inflammation | PIK3R2 | Regulatory subunit of phosphoinositide-3-kinase (PI3K), a kinase that phosphorylates PtdIns(4,5)P2 (Phosphatidylinositol 4,5- bisphosphate) to generate phosphatidylinositol 3,4,5-trisphosphate (PIP3). PIP3 plays a key role by recruiting PH domain-containing proteins to the membrane, including AKT1 and PDPK1, activating signaling cascades involved in cell growth, survival, proliferation, motility and morphology. |
| --- | --- | --- |
|  | PKIA | Extremely potent competitive inhibitor of cAMP-dependent protein kinase activity, this protein interacts with the catalytic subunit of the enzyme after the cAMP-induced dissociation of its regulatory chains. |
|  | POU5F1 | Transcription factor that binds to the octamer motif (5'- ATTTGCAT-3'). |
|  | RAD51B | Involved in the homologous recombination repair (HRR) pathway of double-stranded DNA breaks arising during DNA replication or induced by DNA-damaging agents. |
|  | RGS14 | Regulates G protein-coupled receptor signaling cascades. |
|  | RGS21 | Inhibits signal transduction by increasing the GTPase activity of G protein alpha subunits thereby driving them into their inactive GDP-bound form. |
|  | RNF39 | May play a role in prolonged long term-potentiation (LTP) maintenance. |
|  | SLC44A2 | exhibits some choline transporter activity; Belongs to the CTL (choline transporter-like) family. |
|  | TRIM31 | Regulator of Src-induced anchorage independent cell growth (By similarity). |
|  | TSFM | Associates with the EF-Tu.GDP complex and induces the exchange of GDP to GTP. |
|  | VMP1 | Stress-induced protein that, when overexpressed, promotes formation of intracellular vacuoles followed by cell death. |
|  | ZFP36L1 | Zinc-finger RNA-binding protein that destabilizes several cytoplasmic AU-rich element (ARE)-containing mRNA transcripts by promoting their poly(A) tail removal or deadenylation, and hence provide a mechanism for attenuating protein synthesis. |

**Supplementary table 2 (continued)**

| Pseudogenes | C6orf10 |  |  |
| --- | --- | --- | --- |
|  | HCG4P3 |  |  |
|  | HCG4P7 |  |  |
|  | HLA-DPB2 |  |  |
|  | HLA-DQB3 |  |  |
|  | HLA-W |  |  |
|  | MCCD1P2 |  |  |
|  | MICC |  |  |
|  | MICD |  |  |
|  | MICF |  |  |
|  | MTCO3P1 |  |  |
|  | RP1-102E24.1 |  |  |
|  | SETP16 |  |  |
|  | TMPOP1 |  |  |
|  | UQCRHP1 |  |  |
|  | ZSCAN12P1 |  |  |
