# Supplementary material for: Genetic susceptibility and causal pathway analysis of eye disorders coexisting in multiple sclerosis
Source: Front Immunol. 2024 Feb 5;15:1337528. doi: 10.3389/fimmu.2024.1337528 (PMC10875133; doi:10.3389/fimmu.2024.1337528)
Supplement: Supplementary file 4 [file Table_3.docx]

**Supplementary table 3** Top10 enriched biological processes of nearest genes of the causal SNPs from four MS datasets that indirectly associated with the immune response and inflammation

| **GeneSet** | **Description** | **Size** | **Overlap** | **Expect** | **EnrichmentRatio** | **pValue** | **FDR** | **Genes** |
| --- | --- | --- | --- | --- | --- | --- | --- | --- |
| GO:0008356 | asymmetric cell division | 20 | 2 | 0.031066 | 64.37826 | 4.31E-04 | 0.36646 | POU5F1;RGS14 |
| GO:0048017 | inositol lipid-mediated signaling | 165 | 3 | 0.256298 | 11.70514 | 0.002043 | 0.706287 | MAPK3;PIK3R2;ZFP36L1 |
| GO:0070849 | response to epidermal growth factor | 48 | 2 | 0.074559 | 26.82428 | 0.002493 | 0.706287 | MAPK3;ZFP36L1 |
| GO:0061919 | process utilizing autophagic mechanism | 473 | 4 | 0.73472 | 5.44425 | 0.005619 | 0.750537 | CLEC16A;MAPK3;PIK3R2;VMP1 |
| GO:0006909 | phagocytosis | 238 | 3 | 0.36969 | 8.114907 | 0.005726 | 0.750537 | MAPK3;ELMO1;PIK3R2 |
| GO:0007492 | endoderm development | 74 | 2 | 0.114946 | 17.39953 | 0.005825 | 0.750537 | POU5F1;ZFP36L1 |
| GO:0010608 | posttranscriptional regulation of gene expression | 486 | 4 | 0.754913 | 5.298622 | 0.006181 | 0.750537 | MAPK3;POU5F1;TSFM;ZFP36L1 |
| GO:0048010 | vascular endothelial growth factor receptor signaling pathway | 87 | 2 | 0.135139 | 14.7996 | 0.007969 | 0.846719 | ELMO1;PIK3R2 |
| GO:0007059 | chromosome segregation | 312 | 3 | 0.484636 | 6.190217 | 0.012008 | 1 | RMI2;PDCD6IP;RGS14 |
| GO:0070371 | ERK1 and ERK2 cascade | 326 | 3 | 0.506382 | 5.92438 | 0.013512 | 1 | MAPK3;RGS14;ZFP36L1 |
